# Job Burnout and Occupational Stressors among Chinese Healthcare Professionals at County-Level Health Alliances

**DOI:** 10.3390/ijerph17061848

**Published:** 2020-03-12

**Authors:** Yan Liu, Li Lu, Wen-Xin Wang, Shou Liu, Hong-Ru Chen, Xiang Gao, Ming-Yu Huang, Yong-Nian Liu, Yan-Ming Ren, Chao-Cai Wang

**Affiliations:** 1 Department of Public Health, Medical College of Qinghai University, Xining 810001, Qinghai, China; qhyxyly@aliyun.com (Y.L.); xin313amazing@163.com (W.-X.W.); liushou2004@aliyun.com (S.L.); hongru823@163.com (H.-R.C.); 2 Health Development Research Center, Medical College of Qinghai University, Xining 810001, Qinghai, China; gaoxiang@qhu.edu.cn (X.G.); liuyongnian@qhu.edu.cn (Y.-N.L.); btyqh@126.com (Y.-M.R.); 3 Team IETO, Bordeaux Population Health Research Center, UMR U1219, INSERM, Université de Bordeaux, 33076 Bordeaux, France; 4 Department of Infection Disease, Qinghai Center for Disease Prevention and Control, Xining 810001, Qinghai, China

**Keywords:** job burnout, occupational stressors, healthcare professional, county-level health alliance, plateau area, China

## Abstract

Background: This study aimed to examine the degrees of job burnout and occupational stressors and their associations among healthcare professionals from county-level health alliances in Qinghai–Tibet Plateau, China. Methods: A cross-sectional study was conducted in county-level health alliances in Qinghai Province, China, in November 2018. The Maslach Burnout Inventory—General Survey and the 38-item Chinese version of the “Scale for occupational stressors on clinicians” were used. Medical staff in four health alliances from two counties were invited to complete the questionnaire. Results: A total of 1052 (age: 34.06 ± 9.22 years, 79.1% females) healthcare professionals were included, 68.2% (95% CI: 65.2–71.0%) of the participants had job burnout symptoms. Occupational stressors had positive associations with moderate (OR = 1.06, 95% CI: 1.05–1.07) and serious (OR = 1.15, 95% CI: 1.13–1.19) level of job burnout. Stressors from vocational interest produced the greatest magnitude of odds ratio (OR = 1.76, 95% CI: 1.62–1.92) for serious degree of burnout, followed by doctor–patient relationship, interpersonal relationship as well as other domains of occupational stressors. Conclusions: Job burnout was very common among healthcare professionals working in Chinese county-level health alliances, different occupational stressors had associations with job burnout. Appropriate and effective policies and measures should be developed and implemented.

## 1. Introduction

Job burnout is a common syndrome in healthcare workers [1], and it has become a sophisticated social issue and a sign of karoshi (death by overwork) for exhausted physicians, especially in China [2]. The prevalence of burnout among physicians varied greatly in studies [3], the overall burnout prevalence ranging from 0% to 80.5% among studies using the Maslach Burnout Inventory (MBI) [4]. According to the survey conducted by the Statistical Information Center of the Ministry of Health in China in 2010, 52.4% of healthcare professionals have job burnout, of which 3.1% have a high degree. A greater level of burnout was correlated with sickness absence, more medical errors, poorer-quality healthcare and reduced patient safety [1,5,6,7]. For healthcare professionals, long-term job-related burnout may also lead to behavioral and psychiatric disorders and poor quality of life [7,8]. 

It has been confirmed that the interaction of job demands and job decision latitude can lead to mental strain [9]. Conscientiousness, openness to experience and stress factors were factors that related to job burnout [10,11,12]. Studies using the MBI indicated that the job stress was strongly correlated with overall occupational burnout [13], and occupational stress was also positively associated with the three dimensionalities of job burnout, i.e., exhaustion, cynicism and professional efficacy [14]. 

In recent years, health alliances have been established throughout China as an important facilitator of a graded diagnosis and treatment system [15]. County-level health alliances refer to alliances comprised of a leading county-level hospital and several township clinics in close geographic proximity. The leading hospitals are responsible for business guidance, personnel training, reasonable allocation of funds from the New Rural Cooperative Medical Scheme to the members and provision of primary medical and health services for demanders. Health alliances could improve the quality of primary care in some aspects by helping to shift the high-quality health resources from tertiary and secondary hospitals to the primary health facilities [16].

Qinghai Province is located in Northwestern China and mostly in the Tibetan Plateau, and the province has long been a multi-ethnic settlement. The number of health workers per thousand population in Qinghai Province was 8.17 in 2016, while the figure on county-level or primary hospitals was the lowest [17]. In a context of health care reform and a changing health care environment, medical staff need to address the new requirements of high-level professional skills and the provision of comprehensive health services, which could produce inevitable job stress and potential burnout.

Therefore, the aim of this study was to examine the degrees of job burnout and occupational stressors and their associations among healthcare professionals from county-level health alliances in Qinghai–Tibet Plateau areas, China. Based on the previous studies, we made the hypotheses that job burnout could be common among the participants and there could be associations between job burnout and some domains of occupational stressors.

## 2. Materials and Methods 

### 2.1. Study Design and Data Collection

This cross-sectional study was carried out in the capital city of Qinghai Province of China, i.e., Xining, in November 2018. Healthcare professionals from four county-level health alliances (2 general health alliances and 2 Chinese traditional health alliances) in Minhe county and Ledu district were invited to participate in this survey. All four leading county-level hospitals in those two counties were included in this study. Township health center was selected by a random sampling method, 18 township health centers were finally included, and the cluster-sampling method was used to recruit participants in each hospital and township health center. Temporary or visiting staff was excluded from this study. 

The formula of the sample size calculation is $N=\frac{Z_{a}^{2}\times P\left(1-P\right)}{d^{2}}$ [18]. According to one review, the prevalence of job burnout among Chinese doctors is 66.5–87.8% [19], and we estimated the percentage of loss to follow-up to be 20%, which reflects a response rate of 80%. The sample size should be a minimum of 242 using the prevalence of 66.5%. 

Pre-established electronic questionnaires with a link were sent to healthcare professionals’ WeChat (a Chinese multi-purpose messaging, social media and mobile payment app) working group by administrative staff from the medical department of each hospital; participants returned the questionnaire after completion. The process followed the principle of anonymity and voluntariness. All the healthcare professionals involved in this survey signed the informed consent form. The study protocol was approved by the ethics committee of the Medical College, Qinghai University (QHMC-2018-00037-PH). The report of this study was in accordance with the Strengthening the Reporting of Observational Studies in Epidemiology (STROBE) guidelines [20].

### 2.2. Assessment 

The questionnaire used in this study consisted of three parts: basic social-demographic characteristics; the Maslach Burnout Inventory—General Survey (MBI-GS); and Chinese version of the Scale for occupational stressors on clinicians.

#### 2.2.1. Basic Social–Demographic Characteristics

Basic social–demographic and working characteristics with regard to sex, marital status, professional group, department, working years, hospital level and hospital category were collected.

#### 2.2.2. Measurement of Burnout

The Maslach Burnout Inventory—General Survey (MBI-GS) (Cronbach’s alpha: 0.818) was used to evaluate job burnout among healthcare professionals [21,22]. The Chinese version has been validated and widely used [23]. The scale consists of three dimensions: emotional exhaustion (EE), cynicism (CY) and reduced professional efficacy (PE). The response of each item is a 7-point Likert scale ranging from 0 = never to 6 = daily, indicating the frequency of experiencing each symptom. All items in the dimension of reduced personal efficacy were reverse-coded. The score was weighted by the following formula to express the level of burnout [24]: 

Total score = 0.4 × emotional exhaustion + 0.3 × cynicism + 0.3 × reduced personal efficacy.

Higher scores indicated a higher level of job burnout. In this study, the level of burnout was classified into 3 categories: no burnout (scores 0–1.49), some burnout symptoms (moderate burnout hereafter) (scores 1.50–3.49) and serious burnout (3.50–6) [24]. 

#### 2.2.3. Occupational Stressors 

The 38-item Chinese version of the Scale for occupational stressors on clinicians with satisfactory psychometric properties (Cronbach’s alpha: 0.879) [25] was used to assess the occupational stressors in this study. It is a 4-point Likert, which has been widely used in Chinese studies [26,27]. This scale is composed of seven domains: organization and management (8 items), vocational interest (8 items), work load (6 items), career development (7 items), interpersonal relationship (3 items), external environment (3 items) and doctor–patient relationship (3 items). Each item ranges from “1 (very inconsistent)” to “4 (very consistent)”. Reverse scoring was adopted for the first seven items of vocational interest to avoid deviation caused by participants’ inertial thinking, and higher scores indicate greater levels of stress [25]. The total score is the sum of the scores of each item, and the score for each domain is the sum score of its each item. The whole scale and details of items for each domain are shown in Supplementary Materials Tables S1 and S2.

### 2.3. Statistical Analysis

Description analyses were expressed using frequency, mean and standard deviation (SD) accordingly. The normal distribution of continuous variables was tested using the Shapiro–Wilk test. The Chi square test, t test, analysis of variance (ANOVA) and Kruskal–Wallis H test were conducted appropriately as the univariate analyses. Multinomial logistic regressions were then preformed to identify the associations between job burnout (Y) and occupational stressors as well as each domain by adjusting variables with significant difference in the univariate analysis. In each model, job burnout was set as the dependent variable, total score of occupational stressors and its each domain were separately set as the independent variables. The data were analyzed using SPSS, version 22.0 (SPSS Inc., Chicago, IL, USA) and the significance level was set at 0.05 (two-tailed).

## 3. Results

### 3.1. Basic Characteristics of the Participants

A total of 1129 questionnaires were distributed and 1052 were collected giving a response rate of 93.2%. The participants were from four leading county-level hospitals and 18 township health centers. The average age of the included healthcare professionals was 34.06 (SD = 9.22) years, and 68.2% (717/1052, 95% CI: 65.2–71.0%) of the participants had moderate or serious degrees of job burnout. Table 1 shows the sociodemographic and working characteristics of healthcare professionals regarding different degrees of occupational burnout. 

### 3.2. Occupational Stressors at Different Degrees of Burnout

The levels of occupational stressors at different degrees of burnout are shown in Table 2. The overall stressors score was 95.99 ± 18.15, and the scores for participants without burnout, with moderate and serious degree of burnout were 84.97 ± 17.26, 99.49 ± 14.93 and 119.25 ± 18.13, respectively. The Shapiro–Wilk test showed the total score of occupational stressor was not normally distributed as well as the score on the domains of organization and management, career development, interpersonal relationship and doctor–patient relationship, Kruskal–Wallis tests were conducted as the univariate analysis for them. Significant differences were found on the three degrees of burnout in all seven domains and overall score of occupational stressors (*p* < 0.001).

### 3.3. Associations between Occupational Stressors and Burnout

Multinomial logistic regression models showed that after adjusting the variables with statistical significance in univariate analyses, the total score of occupational stressors and every domain were respectively and positively associated with moderate and serious level of burnout. The odds ratios of total occupational stressors for moderate burnout and serious level of burnout were 1.06 (95% CI: 1.05–1.07) and 1.15 (95% CI: 1.13–1.19), respectively. Occupational stressors from vocational interest (OR = 1.76, 95% CI: 1.62–1.92), doctor–patient relationship (OR = 1.58, 95% CI: 1.35–1.85) and interpersonal relationship (OR = 1.54, 95% CI: 1.35–1.76) showed the highest magnitude of odds ratio to serious level of burnout. Stressors from interpersonal relationship (OR =1.35, 95% CI: 1.25–1.45) and vocational interest (OR = 1.30, 95% CI: 1.25–1.35) showed the highest magnitude of odds ratio to moderate level of burnout (Table 3).

## 4. Discussion

This is the first study examining the degree of job burnout, occupational stressors and their relationship among healthcare professionals from county-level health alliances in Qinghai–Tibet Plateau areas, China. The prevalence of moderate and serious degrees of job-related burnout was 68.2% among our participants. There were positive associations between occupational stressors and job burnout, and vocational interest showed the highest magnitude of odds ratio to serious level of job burnout among the seven domains of occupational stressors, followed by doctor–patient relationship, interpersonal relationship and other domains.

The prevalence of job-related burnout was higher than the figure of 56.6% found in one Chinese study among obstetricians and pediatricians working in provincial hospitals [28], while it was lower than the 76.9% in another Chinese study including physicians from 10 provinces in China [29]. Compared to other countries, our figure was also significantly higher than that among French physicians (49%, 95% CI: 45–53%) [30] and Iranian nurses (36%, 95% CI: 20–53%) [31], and greater than the health professionals in Ecuador (2.6%) [32] and physicians in USA (56.6%) [33]. Inconsistencies on the definition of burnout and assessment tools, etc., could partly explain the disparities between studies [4]. Besides, cultural, regional and economic factors in different countries could have a role; for example, lower annual income was reported to be associated with higher level of burnout [34]. 

This study was conducted among the county-level health alliances, in which the secondary hospital is the leading hospital and the primary hospital is its subordinate. Under the assistance and management of the leading hospital, primary hospitals are mainly responsible for providing healthcare services to the local residents, which means that professionals who work in the primary hospitals would have to enhance their knowledge and acquire more advanced medical techniques to meet the demands for health services [35], which could partly increase their occupational stress and job burnout. 

Occupational stressors were positively associated with both moderate and serious degrees of job burnout, which have been identified in previous studies [36,37]. It has been reported that occupational stressors make the greatest contribution to nurses’ job burnout compared with socio-demographic characteristics [38]. The participants in this study were from county-level health alliances in Qinghai Province, China, which has a relatively lagging economy (compared to some other Provinces in China) and a sparsely distributed population. In 2014, the number of health technicians and practicing (and assistant) physicians per thousand population in Qinghai Province was highest in the capital city, Xining (9.69 vs. 3.44), and lowest in Haidong city (2.65 vs. 0.93) [39]. The implementation and development of the health alliances is a key to deepening the health reform in China. There are still deficiencies in the allocation of primary health care personnel, such as unbalanced distribution and loss of personnel, which could partly result in the extension of working hours and stress of primary healthcare professionals as well as their job burnout.

Vocational interest produced odds ratio of the greatest magnitude to serious job burnout, followed by doctor–patient relationship, interpersonal relationship and other domains of occupational stressors. People will achieve better job performance and obtain a longer term job satisfaction if their interests match their careers [40], and job performance has been frequently reported to have a negative relationship with job burnout [41,42]. The potential mediating role of job performance could also partly explain the strong association between vocational interest and job burnout. China has witnessed a surge in medical disputes in recent years, the detrimental influences of the damaged doctor–patient relationship irresistibly emerged [43]. Besides, the high prevalence of workplace violence from patients and their family or their co-workers has been frequently reported among Chinese healthcare professionals [44], which could also explain the associations between interpersonal relationship and doctor–patient relationship. Stressors from career development and workload also played roles in the high level of burnout. The structural imbalance of healthcare professional titles is prominent in China, and there were great urban–rural and regional gaps [45], which were serious in Qinghai Province. It is difficult for many clinicians, especially young doctors, to be promoted under the current promotion systems, which could definitely influence clinicians’ enthusiasm and lead to psychological disorders. Heavy workload of hospital workers is also a major problem for the Chinese health care system [29].

Healthcare professionals with high burnout were at a greater risk of suffering from poor physical, cognitive and emotional well-being [37,46]. Improving the quality of primary care, physical and mental health of health care providers should also be of concern. It has been identified that both individual-focused and structural or organizational strategies can bring about clinically meaningful reductions in physicians’ burnout [47], especially adoption of organization-directed approaches [48]. Therefore, effective measures to reduce job burnout among medical staff, in particular those measures based on health care organization, should be considered. 

There were some limitations in this study. First, information such as personal economic status, family support and information on the hospitals in which the respondent works, etc., which could be confounding factors were not collected. Second, only healthcare professionals working in county-level health alliances in Qinghai Province were included; participants from multiple provinces should be recruited in future research. In addition, the existing literatures using the Chinese version of the Scale for occupational stressors on clinicians were only published in Chinese, which limited the possible comparisons among studies for international readerships. Lastly, the causal relationship between occupational stressors and job burnout cannot be generated due to the cross-sectional design; thus, further longitudinal studies are warranted.

## 5. Conclusions

In conclusion, job burnout was very common among the healthcare professionals working in the Chinese county-level health alliances. Occupational stressors had positive associations with moderate and serious levels of job burnout. Effective policies and measures should be appropriately developed and implemented at the national, regional or institutional level, accordingly, to alleviate the level of work pressure and job burnout and ensure psychological health of health service providers in Chinese county-level health alliances.

## Figures and Tables

**Table 1:** Basic characteristics of the included participants.

Variables	Categories	*N* (1052)	Degree of Burnout (*n*, %)			*p* Value
			No (*n* = 335)	Moderate (*n* = 657)	Serious (*n* = 60)	
Sex	Male	220	59 (17.6)	139 (21.2)	22 (36.7)	**0.009**
	Female	832	276 (82.4)	518(78.8)	38 (63.3)	
Marital status	Married	778	250 (74.6)	486 (74.0)	42 (70.0)	0.593
	Unmarried	274	85 (25.4)	171 (26.0)	18 (30.0)	
Professional group	Doctors	311	95 (28.4)	189 (28.8)	27 (45.0)	**0.017**
	Nurses	429	124 (37.0)	282 (42.9)	23 (38.3)	
	Medical technicians	199	67 (20.0)	127 (19.3)	5 (8.3)	
	Others	113	49 (14.6)	59 (9.0)	5 (8.3)	
Department	Physical	228	62 (18.5)	153 (23.3)	13 (21.7)	**<0.001**
	Surgical	116	27 (8.1)	77 (11.7)	12 (20.0)	
	Obstetrics and gynecology	123	44 (13.1)	69 (10.5)	10 (16.7)	
	Emergency	88	14 (4.2)	65 (9.9)	9 (15.0)	
	Medical technology	202	74 (22.1)	121 (18.4)	7 (11.7)	
	TCM	295	114 (34.0)	172 (26.2)	9 (15.0)	
Working years	≤5	460	153 (45.7)	284 (43.2)	23 (38.3)	0.191
	~6	237	63 (18.8)	159 (24.2)	15 (25.0)	
	~11	157	50 (14.9)	94 (14.3)	13 (21.7)	
	≥21	198	69 (20.6)	120 (18.3)	9 (15.0)	
Hospital level	Secondary	931	286 (85.4)	590 (89.8)	55 (91.7)	**0.028**
	Primary	121	49 (14.6)	67 (10.2)	5 (8.3)	
Hospital category	General	685	209 (62.4)	428 (65.1)	48 (80.0)	0.053
	TCM	367	126 (37.6)	229 (34.9)	12 (20.0)	

Bold values: *p* < 0.05. TCM: Traditional Chinese medicine.

**Table 2:** Score of occupational stressors at different degrees of burnout among healthcare professionals.

Occupational Stressors (M ± SD)	Total (*N* = 1052)	Degree of Burnout			*p* Value
		No (*n* = 335)	Moderate (*n* = 657)	Serious (*n* = 60)	
Organization and management *	19.61 ± 5.26	17.49 ± 5.55	20.27 ± 4.65	24.33 ± 5.26	<0.001
Vocational interest ^#^	17.59 ± 5.29	13.81 ± 4.40	18.85 ± 4.35	24.83 ± 5.05	<0.001
Workload ^#^	17.23 ± 4.11	16.03 ± 4.24	17.54 ± 3.83	20.48 ± 4.01	<0.001
Career development *	17.79 ± 4.96	15.65 ± 5.15	18.50 ± 4.35	22.10 ± 5.17	<0.001
Interpersonal relationship *	5.42 ± 2.13	4.59 ± 1.96	5.75 ± 2.02	6.48 ± 2.72	<0.001
External environment ^#^	9.49 ± 2.16	9.16 ± 2.14	9.56 ± 2.12	10.62 ± 2.28	<0.001
Doctor–patient relationship *	8.86 ± 2.32	8.24 ± 2.51	9.03 ± 2.14	10.40 ± 2.12	<0.001
Total score *	95.99 ± 18.15	84.97 ± 17.26	99.49 ± 14.93	119.25 ± 18.13	<0.001

^#^ Analysis of variance (ANOVA); * Kruskal–Wallis test.

**Table 3:** The associations between different domains of occupational stressors and burnout.

Occupational Stressors	Moderate Burnout	Serious Burnout
	OR (95% CI)	OR (95% CI)
Organization and management	1.11 (1.08, 1.14) ***	1.30 (1.22, 1.38) ***
Vocational interest	1.30 (1.25, 1.35) ***	1.76 (1.62, 1.92) ***
Workload	1.09 (1.06, 1.13) ***	1.34 (1.22, 1.47) ***
Career development	1.13 (1.10, 1.17) ***	1.34 (1.25, 1.44) ***
Interpersonal relationship	1.35 (1.25, 1.45) ***	1.54 (1.35, 1.76) ***
External environment	1.08 (1.01, 1.15) *	1.42 (1.19, 1.68) ***
Doctor–patient relationship	1.15 (1.08, 1.21) ***	1.58 (1.35, 1.85) ***
Total score	1.06 (1.05, 1.07) ***	1.15 (1.13, 1.19) ***

Note: Multinomial logistic regression models by adjusting for sex, professional group, department, hospital level. Dependent variable: burnout; independent variable in each model: total score of each domain of occupational stressor and the total score, separately. OR: odds ratio, by setting participants without burnout as the reference group. CI: Confidence interval. * *p* < 0.05; *** *p* < 0.001.

## Supplementary Materials

The following are available online at https://www.mdpi.com/1660-4601/17/6/1848/s1, Table S1: The Chinese version of Scale for occupational stressors on clinicians, Table S2: Details of items in each domain in the Scale for occupational stressors on clinicians.
