# Supplementary material for: Job Burnout and Occupational Stressors among Chinese Healthcare Professionals at County-Level Health Alliances
Source: Int J Environ Res Public Health. 2020 Mar 12;17(6):1848. doi: 10.3390/ijerph17061848 (PMC7142970; doi:10.3390/ijerph17061848)
Supplement: Supplementary file 1 [file ijerph-17-01848-s001.pdf]

Supplemental Table S1 The Chinese version of “Scale for occupational stressors on clinicians”.

		Very inconsistent	Somewhat inconsistent	Somewhat consistent	Very consistent
1	Heavy workload and few breaks.				
2	Promotion is slow or has little opportunity for advancement.				
3	I always feel that I do not have time to accompany family and do what I want to do.				
4	Personal schedule was often affected by unexpected situations such as changes in patient's condition, etc.				
5	I really like being a doctor.				
6	Less space for personal development.				
7	Overtime is often required on holidays or weekends.				
8	I am still willing to work in my current unit If I have to choose again.				
9	Functional department is inefficient.				
10	Taking a lot of risk at work.				
11	Currently, there are too many negative reports related to medical staff in the media.				
12	There were difficulties to communicate with co-workers.				
13	Working conditions are very satisfying.				
14	It is difficult to satisfy patients and their families even if you work hard.				
15	Unreasonable and demanding requirements from patients or family members are often encountered.				
16	Alienation with colleagues and it is difficult to integrate with each other.				
17	Rarely get support and help when faced with work difficulties.				
18	I am very interested in my job.				
19	It is difficult to get new knowledge and skills at work				
20	Work performance is difficult to be recognized.				
21	I can't make full use of my abilities at work.				
22	I am satisfied with the work arrangement of the leader.				
23	The relevant employment system of the technical title of the unit is not reasonable.				
24	I'm willing to continue my current job even if I				

	don't run out of money.				
25	The workplace has unreasonable policies, regulations and procedures.				
26	It is difficult to get the understanding and respect from patients and their families.				
27	The workplace lacks effective incentive policies.				
28	I feel that my salary is far lower than what I have devoted.				
29	I rarely get training or instruction.				
30	Being satisfied with the current professional and social status.				
31	Lack of belonging to the workplace.				
32	I rarely involved in the development of organizational systems.				
33	Most colleagues are unfriendly.				
34	Lack of an effective vacation system.				
35	I often work night shifts.				
36	I think the current laws and regulations are difficult to protect the legitimate rights and interests of healthcare professionals.				
37	It is very difficult to get a practical appointment even if I work hard to obtain a higher level of job title.				
38	I think the current healthcare systems need to be improved				

Supplemental Table S2 Details of items in each domain

No.	Domains	Number of items
1	Organization and management	9,23,25,27,28,32,34,37
2	Vocational interest	5,8,13,18,22,24,30 (Reverse scoring for The first seven items require reverse scoring), 31
3	Workload	1,3,4,7,10,35
4	Career development	2,6,17,19,20,21,29
5	Interpersonal relationship	12,16,33
6	External environment	11,36,38
7	Doctor–patient relationship	14,15,26
